# Characterisation of acute respiratory infections at a United Kingdom paediatric teaching hospital: observational study assessing the impact of influenza A (2009 pdmH1N1) on predominant viral pathogens

**DOI:** 10.1186/1471-2334-14-343

**Published:** 2014-06-19

**Authors:** Emily A Lees, Enitan D Carrol, Christine Gerrard, Fiona Hardiman, Gareth Howel, Alison Timmis, Kent Thorburn, Malcolm Guiver, Paul S McNamara

**Affiliations:** 1 Wolfson Centre for Personalised Medicine, University of Liverpool, Block A: Waterhouse Building, 1-5 Brownlow Street, Liverpool L69 3GL, England; 2 Institute of Infection and Global Health, University of Liverpool, The Ronald Ross Building, 8 West Derby Street, Liverpool L69 7BE, England; 3 Microbiology Department, Alder Hey Children’s NHS Foundation Trust Hospital, Eaton Road, Liverpool L12 2AP, England; 4 Hull Royal Infirmary, Anlaby Road, Hull HU3 2JZ, England; 5 Department of Paediatrics, Countess of Chester NHS Foundation Trust, Liverpool Road, Chester CH2 1UL, England; 6 Paediatric Intensive Care Unit, Alder Hey Children’s NHS Foundation Trust Hospital, Eaton Road, Liverpool L12 2AP, England; 7 Molecular Diagnostics Department, Manchester Royal Infirmary, Oxford Road, Manchester M13 9WZ, England; 8 Institute of Child Health, Alder Hey Children’s NHS Foundation Trust Hospital, Eaton Road, Liverpool L12 2AP, England

**Keywords:** H1N1, Influenza, Paediatrics, Virology, Co-morbidities

## Abstract

**Background:**

According to the World Health Organisation, influenza A (2009 pdmH1N1) has moved into the post-pandemic phase, but there were still high numbers of infections occurring in the United Kingdom in 2010-11. It is therefore important to examine the burden of acute respiratory infections at a large children’s hospital to determine pathogen prevalence, occurrence of co-infection, prevalence of co-morbidities and diagnostic yield of sampling methods.

**Methods:**

This was a retrospective study of respiratory virus aetiology in acute admissions to a paediatric teaching hospital in the North West of England between 1st April 2010 and 31st March 2011. Respiratory samples were analysed either with a rapid RSV test if the patient had symptoms suggestive of bronchiolitis, followed by multiplex PCR testing for ten respiratory viruses, or with multiplex PCR testing alone if the patient had suspected other ARI. Patient demographics and data regarding severity of illness, presence of co-morbidities and respiratory virus sampling method were retrieved from case notes.

**Results:**

645 patients were admitted during the study period. 82/645 (12.7%) patients were positive for 2009 pdmH1N1, of whom 24 (29.2%) required PICU admission, with 7.3% mortality rate. Viral co-infection occurred in 48/645 (7.4%) patients and was not associated with more severe disease. Co-morbidities were present more frequently in older children, but there was no significant difference in prevalence of co-morbidity between 2009 pdmH1N1 patients and those with other ARI. NPA samples had the highest diagnostic yield with 192/210 (91.4%) samples yielding an organism.

**Conclusions:**

Influenza A (2009 pdmH1N1) is an ongoing cause of occasionally severe disease affecting both healthy children and those with co-morbidities. Surveillance of viral pathogens provides valuable information on patterns of disease.

## Background

Acute respiratory infection (ARI) is an important cause of death worldwide [1]. The recent influenza A (2009 pdmH1N1) pandemic contributed significantly to morbidity and mortality from ARI [2]. In the 2009-10 season, the United Kingdom (UK) experienced 800,000 2009 pdmH1N1 infections and 457 deaths, and spent £1.2 billion on treatment and prevention campaigns [3]. In August 2010, the Director General of the World Health Organisation declared that the virus was in the post-pandemic phase and likely to be circulating for years to come [4].

Despite weekly updates on the Public Health England website about the numbers of circulating cases of influenza and some other viruses [5], there is limited published data on which respiratory viral pathogens cause ARI in children in the UK. Our study has attempted to address this knowledge gap by describing viral pathogen prevalence, occurrence of co-infection, diagnostic yield of sampling methods and presence of co-morbidity in patients with ARI caused by 2009 pdmH1N1 and other respiratory viruses, in a large paediatric teaching hospital in the North West of England over a year between April 2010 and March 2011.

## Methods

### Setting and study design

This was a retrospective study of the case notes of patients with suspected ARI who had respiratory samples sent for rapid respiratory syncytial virus (RSV) testing and respiratory virus PCR analysis at Alder Hey Children’s NHS Foundation Trust between 1st April 2010 and 31st March 2011. Diagnosis of ARI was based on clinical examination by admitting doctor and case notes were reviewed to confirm coded diagnosis. Alder Hey is a large, university-affiliated paediatric teaching hospital with a catchment area of 7.5 million people and more than 270,000 patient care episodes per annum, including 65,000 children seen in the emergency department [6].

### Subjects

#### Inclusion criteria

All children aged 0–16 years, symptomatic of ARI from whom respiratory virus samples were taken, either at presentation to hospital or within 7 days of admission. Patients who had been admitted for surgery but developed PCR-positive ARI within 7 days of admission were included, as this time frame incorporates the incubation period for all of the viruses studied [7-9]. Data for these patients are highlighted as they were initially elective admissions and thus may display different clinical characteristics to those for whom ARI was the primary cause of admission. Data was collected on clinical characteristics of the ARI including: disease severity, presence of any co-morbidities and length of hospital stay. Co-morbidities were recorded in the categories of: haematology/oncology, respiratory, cardiac, neurological, congenital/chromosomal and other (including gastrointestinal and rheumatological conditions).

#### Exclusion criteria

Children from whom samples were taken more than 7 days after hospital admission, as it was considered that these would be due to nosocomial rather than community-acquired infection.

Definitions of disease severity:

*Mild:* No oxygen requirement

*Moderate:* Oxygen requirement but not requiring admission to Paediatric Intensive Care unit (PICU)

*Severe:* Oxygen requirement and requiring admission to PICU

#### Ethics statement

This study was approved as an audit by the IRB board at the study hospital.

### Pathogen detection

A number of sampling methods were used: nose/throat (N/T) swabs, nasopharyngeal aspirate (NPA), endotracheal aspirate (ETA), bronchoalveolar lavage (BAL) and sputum. Remel MicroTest M4RT was the transport medium used (Oxoid, Basingstoke, United Kingdom). The type of sample collected was at the discretion of clinical staff. Respiratory samples were analysed in one of two ways; either with a rapid RSV test if the patient had symptoms suggestive of bronchiolitis, followed by multiplex PCR testing for ten respiratory viruses, or with multiplex PCR testing alone if the patient had suspected other ARI.

#### RSV testing

This was completed on-site, using BinaxNOW RSV kit (Alere Ltd, Stockport, United Kingdom) according to the manufacturer’s instructions. The test is performed on nasopharyngeal aspirates and detects RSV fusion protein antigen.

#### Respiratory virus PCR analysis

The respiratory PCR screening assay comprises 5 multiplex reactions detecting a total of 10 respiratory viruses. The multiplex reactions detect influenza A (CDC published protocol [10]) and influenza B, respiratory syncytial virus and human metapneumovirus [11], adenovirus and human rhinovirus [12], parainfluenza type 1, 2 and 3 and a specific assay for the detection of the 2009 pdmH1N1 based on a National Standard Method developed by the Health Protection Agency Microbiology Services. The 2009 pdmH1N1 assay was done if initial PCR was positive for influenza A. The 2009 pdmH1N1 assay was prepared in a lyophilised format by Life Technologies (Invitrogen, Paisley, United Kingdom), and all other assays were performed with the use of a SuperScript III Platinum one-step qRT-PCR kit (Invitrogen) in a reaction volume of 30 μl. Thermal cycling was carried out using a Life Technologies 7500 instrument at 50°C for 30 minutes and at 95°C for 2 minutes, followed by 45 cycles of denaturation at 95°C for 15 seconds and annealing and extension at 60°C for 1 minute. Respiratory virus PCR assays were performed at the Public Health Laboratory, Manchester. From December 2010 onwards, assays for 2009 pdmH1N1 were performed on site at the study hospital. A full evaluation of the method using a range of titres for positive and negative controls and dual testing of patient samples between the two centres was undertaken prior to commencing the assays at the study hospital to ensure comparable results between sites.

### Statistical analysis

All results were analysed using Microsoft Excel 2010. Differences in outcomes were examined using Chi squared test, with a p value <0.05 considered significant. Where data were non-parametrically distributed, a Kruskal-Wallis test was used.

## Results

### Demographics and patient characteristics

**Figure 1:**
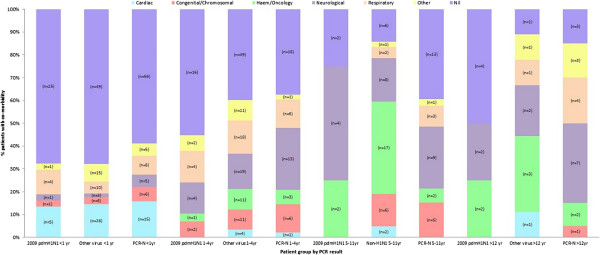
Co-morbidities in 2009 pdmH1N1 admissions versus admissions positive for other viruses or PCR-negative (PCR-N).

The 645 admissions with ARI in this study represented 4% of 17,150 acute medical admissions between 1st April 2010 and 31st March 2011. Median (range; IQR) patient age was 0.9 (0–16; 0.3 – 3.3) years with 238/645 (36.9%) children being < 1 year. 612/645 patients (94.9%) presented with suspected acute respiratory infection and 33/645 (5.1%) became symptomatic with ARI within seven days of admission for surgery.Overall, 158/645 patients (24.5%) were classified as having mild disease, 229/645 (35.5%) moderate and 258/645 (40%) severe. Of those with ARI following surgery, 16/33 (48.5%) were mild, 2/33 (6%) moderate and 15/33 (45.5%) severe. Median (range; IQR) length of stay was 6 (0–339; 2–11) days and 369 patients (65.5%) with ARI had a co-morbidity (see Figure 1). 21/645 (3.3%) patients died during the study period.

### Types of respiratory sample

**Figure 2:**
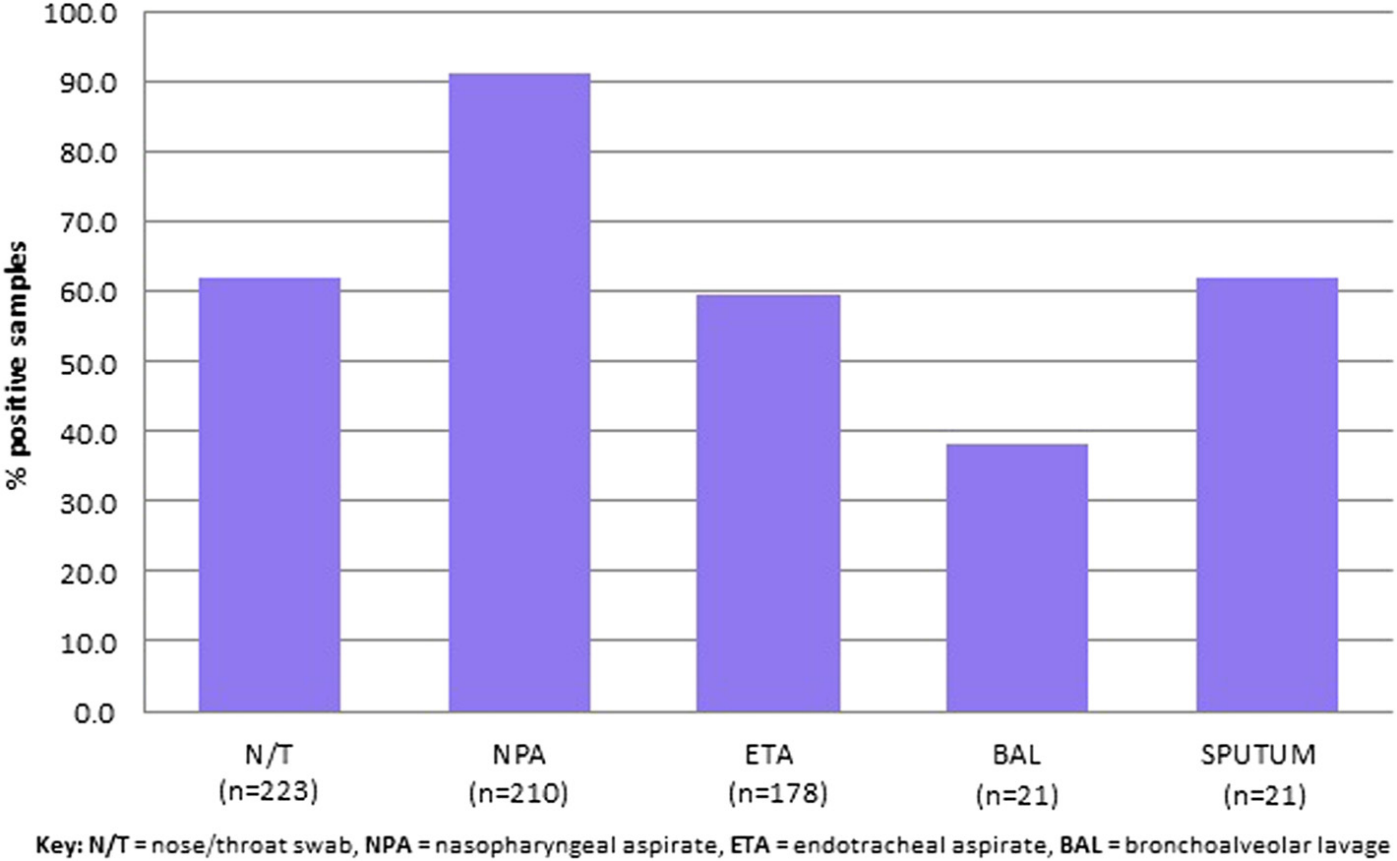
Percentage of positive samples by sampling method.

Six hundred and fifty-three respiratory samples were collected from 645 children. Eight patients had repeat sampling within 4 days of their original sample which detected an additional respiratory virus. The most common sampling methods used were NPA and N/T swab (Figure 2). Of these, NPA samples had the highest diagnostic yield, with a virus being detected in 192/210 (91.4%) samples compared to 138/223 (61.9%) of N/T samples (P < 0.001).

### Pathogens causing ARI

A respiratory virus was detected in 503/653 samples (77%) taken. 450/503 (89.5%) positive samples were taken within 48 hours of admission. Of these samples, 426/450 (94.7%) were taken within the first 24 hours of admission.

The commonest organism detected was RSV (found in 196/503 (39%) positive samples), followed by 2009 pdmH1N1 (82/503; 16.3%) and hRV (human rhinovirus) (79/503; 15.7%). Co-infection occurred in 48/645 patients (7.4%) (Table 1). The pathogen most commonly identified in co-infections was adenovirus; co-infection was detected in 24/49 patients with adenovirus (20 dual, 3 triple and 1 quadruple co-infection). RSV and 2009 pdmH1N1 co-infection occurred in 9 cases (Additional file 1). Co-infection was not associated with disease severity; percentages of patients with mild, moderate and severe disease were comparable for infection with 1 versus 2 viruses (28%, 39% and 33% respectively compared to 28%, 37% and 33%). PICU admission was significantly more likely to occur in patients with PCR-negative samples (110/196; 56%) than those in whom 1 (132/401; 33%), 2 (15/43; 35%) or 3 (0/4; 0%) viruses were detected (p < 0.001).

**Table 1:** Pathogens detected in single and co-infections during the study period

**Pathogen**	**No. single infections**	**No. occurrences in co-infections**	**Total no. occurrences**	**% occurrences as co-infector**
Adenovirus	25	24	49	49.0
Influenza A (non-2009 pdmH1N1)	4	3	7	42.9
Influenza A 2009 pdmH1N1	69	13	82	15.9
Influenza B	30	9	39	23.1
Human metapneumovirus	21	5	26	19.2
Parainfluenza 1	4	1	5	25.0
Parainfluenza 2	1	0	1	0
Parainfluenza 3	15	4	19	21.1
Human rhinovirus	64	15	79	19.0
Respiratory syncytial virus	168	28	196	14.3
Total	401	102	503	

### 2009 pdmH1N1 infection

2009 pdmH1N1 infection was detected in 82/645 children (12.7%), with median (range; IQR) patient age 1.3 (0.1-15; 0.6 – 3.5) years and 37/82 (45.1%) children being under 1 year of age.

Median (range; IQR) length of stay for admissions with 2009 pdmH1N1 infection was 4 days (0–56; 2–11), in comparison to 6 days for all other admissions studied (0–339; 3–11 days). Disease severity was classed as mild in 30/82 (36.6%) 2009 pdmH1N1 patients, moderate in 28/82 (34.1%), and severe in 24/82 (29.2%) cases. As expected, patients with severe disease had significantly longer median hospital stay (12.5 days), than patients who only required general medical ward care (3 days) (p = 0.02). The median (range; IQR) length of time spent in PICU for 2009 pdmH1N1 patients was 5.5 days (1–54; IQR 3–16).

### Prevalence of co-morbidities in patients with 2009 pdmH1N1 infection

Figure 1 shows the prevalence of different co-morbidities in children infected with 2009 pdmH1N1, versus those who had any other respiratory virus and PCR-negative patients based on age.

In children < 1 yr, the prevalence of co-morbidities was similar (2009 pdmH1N1 32%, other respiratory virus 32%, PCR-negative 41%), with congenital cardiac and respiratory (most frequently chronic lung disease of prematurity) conditions being most prevalent across the three groups.

In children aged 1-5 yrs, 2009 pdmH1N1 patients had the lowest incidence of existing co-morbidities (55%), compared to those with other respiratory virus (61%) and PCR-negative (62.5%) admissions (p > 0.05). Neurological and respiratory conditions were the most frequent co-morbidities across all admissions.

In children aged 5-12 yrs, co-morbidities were very common, with patients with other respiratory viruses having the highest frequency of co-morbidities (86%), followed by 2009 pdmH1N1 (75%) and PCR-negative (61%) (p < 0.05). Neurological co-morbidities were again common across all groups, with the highest incidence occurring in 2009 pdmH1N1 admissions. Haematology/oncology conditions were also common in both 2009 pdmH1N1 and admissions with other respiratory viruses.

In >12 yr olds, co-morbidities were again very frequent overall, being found in 85% PCR-negative and 89% other respiratory virus admissions. 2009 pdmH1N1 admissions had the lowest rate of co-morbidity here (50%) (p > 0.05). As with the 5-12 yr cohort, neurological conditions were frequent in all groups with haematological/oncological conditions again being common in 2009 pdmH1N1 and patients with other respiratory viruses.

Overall, co-morbidities became more prevalent with increasing age of the patient, but there was no significant difference in levels of co-morbidity between patients with 2009 pdmH1N1, other respiratory virus and PCR-negative patients.

### Disease severity in relation to infecting virus

**Figure 3:**
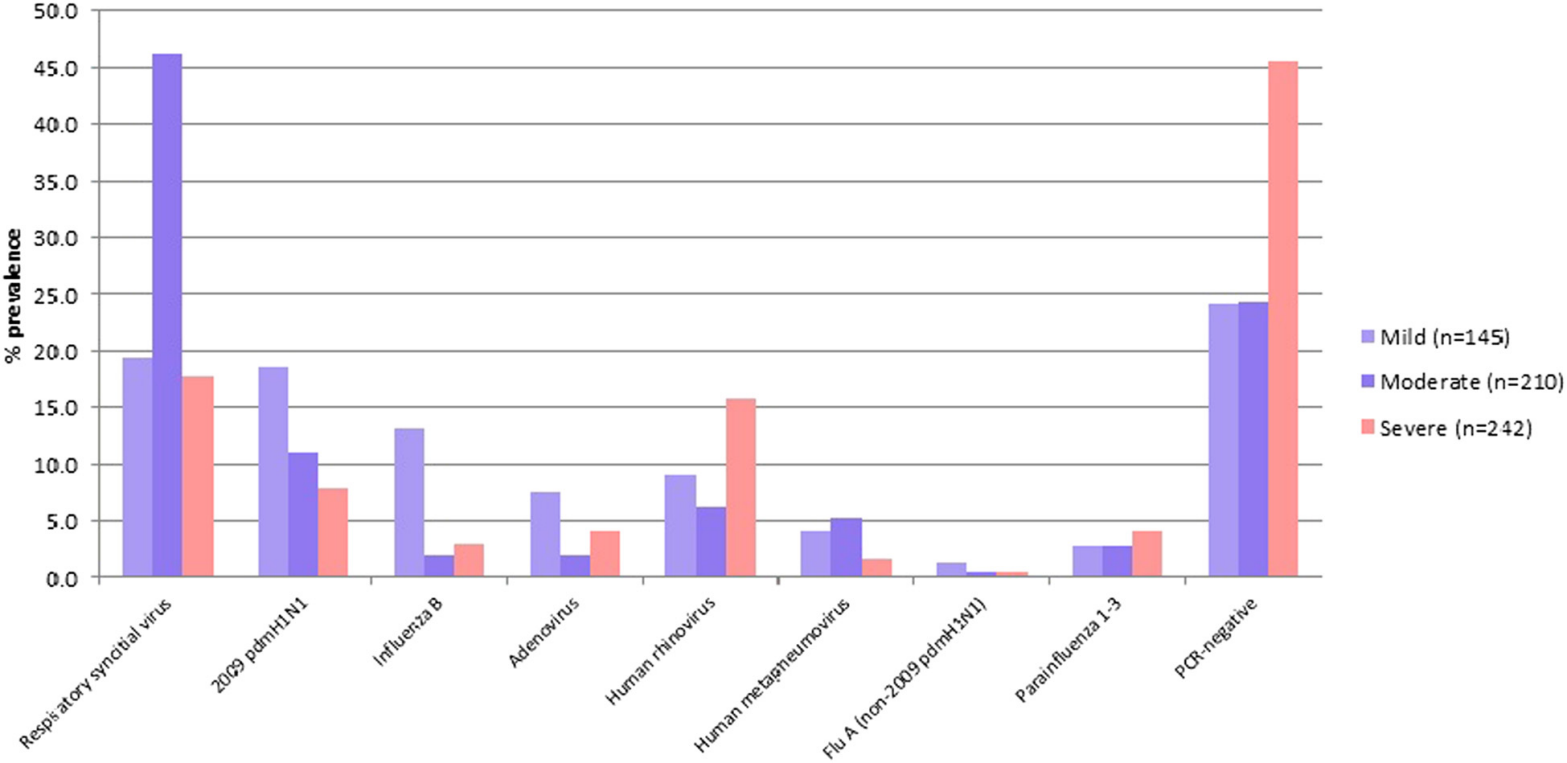
Percentage prevalence of disease severity for each pathogen.

There was a significant difference in mortality between RSV admissions (1%; 2/196 patients) and 2009 pdmH1N1 admissions (7.3%; 6/82 patients) (p < 0.01). Mortality rates during the period studied were 4.6% for PCR-negative admissions (9/196 patients) and 5.1% for influenza B (2/39 patients). There were no fatalities with non-2009 pdmH1N1 influenza A.All viruses were detected in all severities of disease. For RSV, moderate disease was most common. For 2009 pdmH1N1, mild disease was most common, and for PCR-negative patients and those with hRV, severe disease was most common (p < 0.05) (Figure 3).

### Seasonality of viruses 2010/11

**Figure 4:**
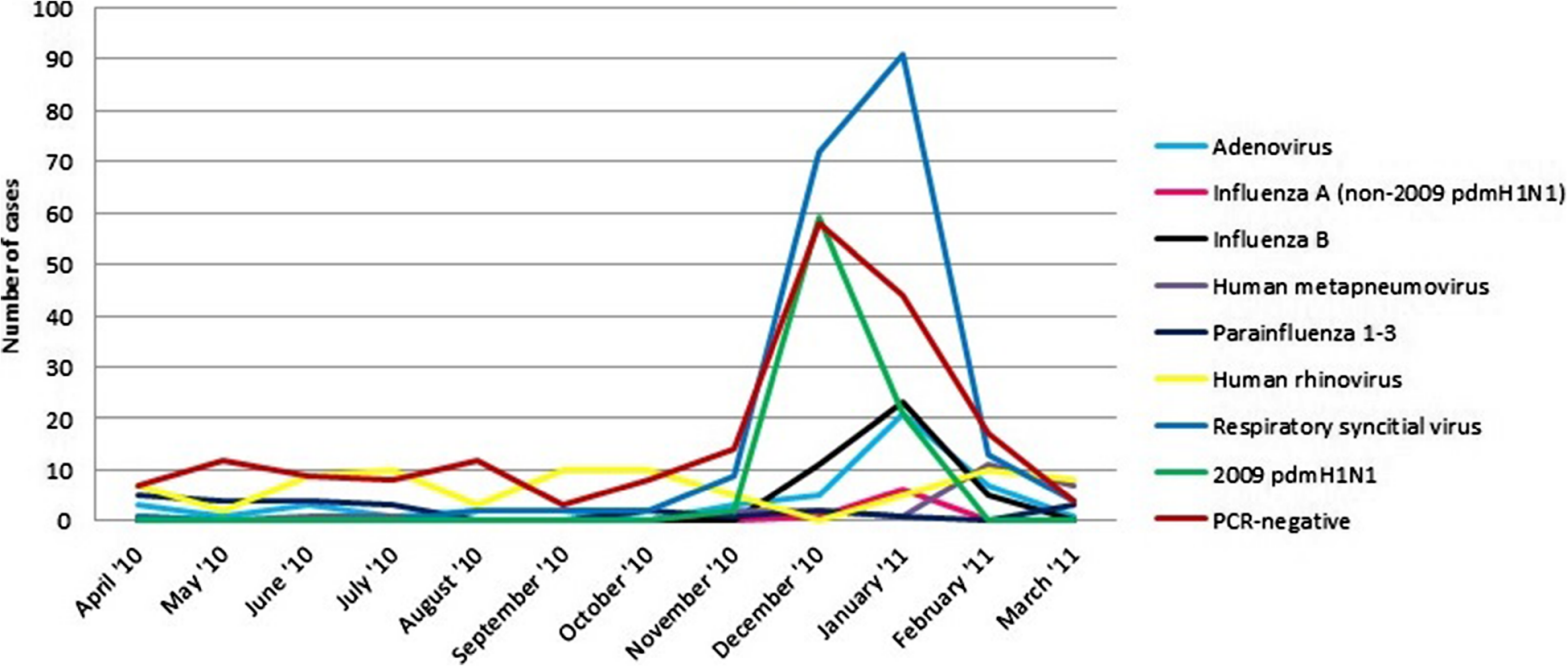
Seasonality of viruses, showing number of cases of each virus per month from April 2010-March 2011.

Post-pandemic 2009 pdmH1N1 seasonality in 2010–11 was very well defined, with all cases (excepting 2 noted in November) occurring during December 2010 and January 2011 (Figure 4).

## Discussion

In this study, 2009 pdmH1N1 caused a spectrum of illness varying from mild to severe disease with mortality both in previously healthy children and those with co-morbidities. Viral co-infection was found in a small proportion of cases and did not appear to be associated with severe disease. We found the prevalence of co-morbidities to be least in children <1 year old, whether infected with 2009 pdmH1N1 or not. Generally, the presence of co-morbidities was more frequent in older cohorts. In younger children, cardiac and respiratory conditions tended to be most frequent, with neurological and haematology/oncology conditions becoming more prevalent in older children.

A study from Birmingham, UK describing experiences during the first wave of 2009 pdmH1N1 cases in 2009 found that 31/77 (40%) patients admitted and all 6 patients who required HDU/ICU care had significant co-morbidities [9]. Similar findings have been reported in a number of other countries [13-16]. In the UK, a study of influenza deaths in 2009–10 by Public Health England found that children with co-morbidities had a higher case fatality rate when infected with 2009 pdmH1N1 compared to seasonal influenza [2]. In our study, two thirds of 2009 pdmH1N1 patients who died had significant co-morbidities, in keeping with UK national data [17].

At the start of the pandemic in August 2009, it was predicted that 3.8% of hospitalised 2009 pdmH1N1 patients under 15 yrs in the UK would require PICU admission [18]. Data published subsequently on the first 2009 pdmH1N1 ‘wave’ showed that 7.7% of infected patients in Birmingham, UK [13], 4.8% in Turin, Italy [15] and 19% in Buenos Aires, Argentina [19] required PICU admission. Mortality rate amongst those with 2009 pdmH1N1 infection in the Argentinean study was 5%, with the majority occurring in patients under 1 year. In contrast, this group in our study had a 2.7% mortality rate, with overall mortality of 7.3% for all 2009 pdmH1N1 admissions.

In our institution, 29% of patients in whom 2009 pdmH1N1 was detected required PICU admission, with median length of stay of 5.5 days. This very high rate may reflect the direct transfer of 2009 pdmH1N1-infected patients into our PICU from other hospitals, as it is a large regional centre. However, this would not account for all of the variation; other causes could include the high number of children with complex medical needs who are under the care of the hospital, a shortfall in HDU beds associated with the 2009 pdmH1N1 pandemic leading to direct PICU admission, or improved virological surveillance of PICU patients leading to a 2009 pdmH1N1 diagnosis.

In our study, Adenovirus was the most common organism found in co-infections; however, co-infection rates in our study were very low compared with international data [20]. This disparity with other studies is likely due in large part to social, environmental and climatic differences between countries. Recent studies have found adenovirus and bocavirus particularly common in co-infections [21]. Both these viruses persist in respiratory secretions for weeks to months, so it is difficult to ascertain whether the presence of these pathogens is due to acute infection or viral persistence within the respiratory tract [20].

We did not find a convincing link between presence of viral co-infection and severity of illness. In fact, PCR-negative patients had the highest rates of PICU admission, suggesting that these patients had bacterial rather than viral illnesses. There appears to be no current consensus on whether viral co-infection is associated with disease severity [22,23]. Of those with ARI following surgery, a comparable proportion to those who attended with suspected ARI were classified as severe. There were however more mild cases in the post-surgical group, who may not otherwise have been hospitalised. In our study, we found that NPA samples had a much higher diagnostic yield for virus detection compared to other sampling methods. This is in keeping with the findings of Chan et al. [24] who found NPA to be the optimal specimen type for diagnosis of viral ARI. However, de la Tabla et al. [25] found N/T swabs to have a higher diagnostic yield, with a combination of both methods providing the highest sensitivity. It is likely that the diagnostic yield for NPAs was so high in our study because most were taken to detect RSV during the bronchiolitis ‘season’ when the ‘hit’ rate was likely to be highest.

### Limitations of study

We were unable to collect information on bacterial culture during the study; this information would have been valuable in assessing whether severe disease in PCR-negative patients, or those with hRV was due to an underlying bacterial infection. We also do not have data on viral load for viruses detected, which could have helped to differentiate between infection and asymptomatic shedding. This study gathered data over the space of one year, incorporation of data from the previous and following years could have provided information on fluctuation of circulating viruses. This is a single site study; however there is no reason to suspect that Liverpool would differ from other UK cities in terms of circulating pathogens. It would have been useful to record influenza immunisation status of admissions, as this may have influenced individuals’ susceptibility to influenza infection and likelihood of hospitalisation.

This study elucidates aspects of ARI experience in the UK, but an unexpected strength is that it raises discussion on current cohorting practices for inpatients. Respiratory viral diagnosis is recommended for all hospitalised infants in order to ascertain disease aetiology and allow optimal use of isolation cubicles to prevent nosocomial infection. Importantly, we have shown that a wide range of viral single infections and co-infections occurred in patients with RSV-negative ARI who would not necessarily have been isolated or cohorted, increasing risk of nosocomial transmission to other patients on the ward [22]. Rapid multiplex respiratory virus testing allows early identification of non-RSV single and co-infections in real time, and enables more effective isolation and cohorting strategies to reduce nosocomial respiratory virus transmission, and allow better utilisation of cubicles.

## Conclusions

Our study found 2009 pdmH1N1 to be the second most common circulating viral pathogen (after RSV) in all patients admitted with suspected ARI between April 2010 and March 2011. Patients with 2009 pdmH1N1 frequently required PICU admission and mortality rates were significantly higher for 2009 pdmH1N1 disease than RSV. Co-morbidities in admitted patients became more prevalent with increasing age.

This study confirms influenza A 2009 pdmH1N1 infection as an ongoing cause of occasionally severe disease affecting both healthy children and those with co-morbidities.

## Competing interests

The authors declare that they have no competing interests.

## Authors’ contributions

EL analysed the data and drafted the manuscript, EC and PM conceived of the study, participated in its design and coordination and helped to draft and revise the manuscript. CG, FH and MG completed the analysis of the samples and CG compiled the results for analysis. GH completed analysis of results from the first part of the study period. AT helped to collect the data for the study. KT helped to draft the manuscript. All authors read and approved the final manuscript.

## Pre-publication history

The pre-publication history for this paper can be accessed here:

http://www.biomedcentral.com/1471-2334/14/343/prepub

## Supplementary Material

Additional file 1 Breakdown of viruses in single and co-infections. Click here for file

## References

[B1] World Health Organisation Global Action Plan for Prevention and Control of Pneumonia (GAPP) November 2009. Available from: http://whqlibdoc.who.int/hq/2009/WHO_FCH_CAH_NCH_09.04_eng.pdf?ua=1

[B2] Pebody RG McLean E Zhao H Cleary P Bracebridge S Foster K Charlett A Hardelid P Waight P Ellis J Bermingham A Zambon M Evans B Salmon R McMenamin J Smyth B Catchpole M Watson JM Pandemic Influenza A (H1N1) 2009 and mortality in the United Kingdom: risk factors for death, April 2009 to March 2010 Euro Surveill 2010 14 20 20504388

[B3] Bosely S Swine flu response was £1.2 billion well spent, review finds Guardian [Internet]. 1 July 2010; Available from: http://www.guardian.co.uk/world/2010/jul/01/swine-flu-response-review-gsk

[B4] Chan M H1N1 in post-pandemic period World Health Organiz 2010 Available from: http://www.who.int/mediacentre/news/statements/2010/h1n1_vpc_20100810/en/

[B5] Health Protection Agency National Influenza Weekly Reports Archive (Update November 3rd 2011) Available from: http://www.hpa.org.uk/web/HPAweb&HPAwebStandard/HPAweb_C/1222154877315

[B6] Alder Hey Children’s NHS Foundation Trust hospital website Available from: http://www.alderhey.nhs.uk

[B7] Aitken C Jeffries DJ Nosocomial spread of viral disease Clin Microbiol Rev 2001 14 3 528 546 10.1128/CMR.14.3.528-546.2001 11432812 PMC88988

[B8] Lessler J Reich NG Brookmeyer R Perl TM Nelson KE Cummings DA Incubation periods of acute respiratory viral infections: a systematic review Lancet Infect Dis 2009 14 291 300 19393959 10.1016/S1473-3099(09)70069-6 PMC4327893

[B9] Degail MA Hughes GJ Maule C Holmes C Lilley M Pebody R Bonnet J Bermingham A Bracebridge S A human metapneumovirus outbreak at a community hospital in England, July to September 2010 Euro Surveill 2012 14 15 22516049

[B10] World Health Organisation CDC protocol of realtime RTPCR for influenza A (H1N1) (revision 2) 2009 Available from: http://www.who.int/csr/resources/publications/swineflu/realtimeptpcr/en/

[B11] Maertzdorf J Wang CK Brown JB Quinto JD Chu M De Graaf M van den Hoogen BG Spaete R Osterhaus AD Fouchier RA Real-time reverse transcriptase PCR assay for detection of human metapneumoviruses from all known genetic lineages J Clin Microbiol 2004 14 3 981 986 10.1128/JCM.42.3.981-986.2004 15004041 PMC356857

[B12] Scheltinga SA Templeton KE Beersma MF Claas EC Diagnosis of human metapneumovirus and rhinovirus in patients with respiratory tract infections by an internally controlled multiplex real-time RNA PCR J Clin Virol 2005 14 4 306 311 10.1016/j.jcv.2004.08.021 15994117 PMC7185544

[B13] Hackett S Hill L Patel J Ratnaraja N Ifeyinwa A Farooqi M Nusgen U Debenham P Gandhi D Makwana M Smit E Welch S Clinical characteristics of paediatric H1N1 admissions in Birmingham, UK Lancet 2009 14 9690 605 19700001 10.1016/S0140-6736(09)61511-7

[B14] Jouvet P Hutchison J Pinto R Menon K Rodin R Choong K Kesselman M Veroukis S Andre Dugas M Santschi M Guerguerian AM Withington D Alsaati B Joffe AR Drews T Skippen P Rolland E Kumar A Fowler R Canadian Critical Care Trials Group H1N1 Collaborative Critical illness in children with influenza A/pH1N1 2009 infection in Canada Pediatr Crit Care Med 2010 14 5 603 609 10.1097/PCC.0b013e3181d9c80b 20308929

[B15] Calitri C Gabiano C Garazzino S Pinon M Zoppo M Cuozzo M Scolfaro C Tovo PA Clinical features of hospitalised children with 2009 H1N1 influenza virus infection Eur J Pediatr 2010 14 12 1511 1515 10.1007/s00431-010-1255-y 20652313

[B16] Lister P Reynolds F Parslow R Chan A Cooper M Plunkett A Riphagen S Peters M Swine-origin influenza virus H1N1, seasonal influenza virus, and critical illness in children Lancet 2009 14 9690 605 607 19700000 10.1016/S0140-6736(09)61512-9

[B17] Sachedina N Donaldson LJ Paediatric mortality related to pandemic influenza A H1N1 infection in England: an observational population-based study Lancet 2010 14 9755 1846 1852 10.1016/S0140-6736(10)61195-6 21030071

[B18] Ercole A Menon DK O’Donnell DR Modelling the impact of pandemic influenza A(H1N1) on UK paediatric intensive care demand Arch Dis Child 2009 14 12 962 964 10.1136/adc.2009.171520 19933604

[B19] Libster R Bugna J Coviello S Hijano DR Dunaiewsky M Reynoso N Cavalieri M Guglielmo M Areso S Gilligan T Santucho F Cabral G Gregorio GL Moreno R Lutz MI Panigasi AL Saligari L Caballero MT Egües Almeida RM Gutierrez Meyer ME Neder MD Davenport MC Del Valle MP Santidrian VS Mosca G Garcia Domínguez M Alvarez L Landa P Pota A Boloñati N Pediatric hospitalizations associated with 2009 pandemic influenza A (H1N1) in Argentina N Engl J Med 2010 14 1 45 55 10.1056/NEJMoa0907673 20032320

[B20] McNamara PS Van Doorn R Cook G Chapter 23 - Respiratory Viruses Manson’s Tropical Diseases 2012 23 London: Elsevier

[B21] Bezerra PG Britto MC Correia JB Duarte Mdo C Fonceca AM Rose K Hopkins MJ Cuevas LE McNamara PS Viral and atypical bacterial detection in acute respiratory infection in children under five years PLoS One 2011 14 4 e18928 10.1371/journal.pone.0018928 21533115 PMC3078930

[B22] Tregoning JS Schwarze J Respiratory viral infections in infants: causes, clinical symptoms, virology, and immunology Clin Microbiol Rev 2010 14 1 74 98 10.1128/CMR.00032-09 20065326 PMC2806659

[B23] Calvo C Pozo F Garcia-Garcia ML Sanchez M Lopez-Valero M Perez-Brena P Casas I Detection of new respiratory viruses in hospitalized infants with bronchiolitis: a three-year prospective study Acta Paediatr 2010 14 6 883 887 10.1111/j.1651-2227.2010.01714.x 20163373 PMC7159545

[B24] Chan KH Peiris JS Lim W Nicholls JM Chiu SS Comparison of nasopharyngeal flocked swabs and aspirates for rapid diagnosis of respiratory viruses in children J Clin Virol 2008 14 1 65 69 10.1016/j.jcv.2007.12.003 18242124

[B25] de la Tabla VO Masia M Antequera P Martin C Gazquez G Bunuel F Gutierrez F Comparison of combined nose-throat swabs with nasopharyngeal aspirates for detection of pandemic influenza A/H1N1 2009 virus by real-time reverse transcriptase PCR J Clin Microbiol 2010 14 10 3492 3495 10.1128/JCM.01105-10 20702662 PMC2953095

