# Supplementary material for: Characterisation of acute respiratory infections at a United Kingdom paediatric teaching hospital: observational study assessing the impact of influenza A (2009 pdmH1N1) on predominant viral pathogens
Source: BMC Infect Dis. 2014 Jun 19;14:343. doi: 10.1186/1471-2334-14-343 (PMC4091667; doi:10.1186/1471-2334-14-343)
Supplement: Additional file 1 — Breakdown of viruses in single and co-infections. [file 1471-2334-14-343-S1.docx]

**Additional file 1: Breakdown of viruses in single and co-infections**

| **Pathogen** | **No. of patients** |
| --- | --- |
| AdV | 25 |
| AdV + FluB | 2 |
| AdV + hMPV | 2 |
| AdV + hRV | 6 |
| AdV + RSV | 8 |
| AdV + 2009 pdmH1N1 | 2 |
| AdV + FluA + RSV | 1 |
| AdV + FluB + RSV | 1 |
| AdV + RSV + 2009 pdmH1N1 | 1 |
| AdV + hMPV + hRV + RSV | 1 |
| Flu A (Non-2009 pdmH1N1) | 4 |
| Flu A + RSV | 1 |
| Flu A + Flu B + RSV | 1 |
| Flu B | 30 |
| Flu B + RSV | 4 |
| Flu B + 2009 pdmH1N1 | 1 |
| hMPV | 21 |
| hMPV + hRV | 2 |
| PCR-negative | 196 |
| PIV1 | 4 |
| PIV1 + hRV | 1 |
| PIV2 | 1 |
| PIV3 | 15 |
| PIV3 + hRV | 4 |
| hRV | 64 |
| hRV + RSV | 1 |
| RSV | 167 |
| RSV + 2009 pdmH1N1 | 9 |
| 2009 pdmH1N1 | 69 |
| **Total** | **645** |

**Key:**

AdV = adenovirus

FluB = influenza B

FluA = influenza A (non-2009 pdmH1N1)

hMPV = human metapneumovirus

hRV = human rhinovirus

PIV1 = parainfluenza virus 1

PIV 2 = parainfluenza virus 2

PIV 3 = parainfluenza virus 3

RSV = respiratory syncytial virus

2009 pdmH1N1 = influenza A 2009 pdmH1N1
